# Feasibility of Observing Glymphatic System Activity During Sleep Using Diffusion Tensor Imaging Analysis Along the Perivascular Space (DTI-ALPS) Index

**DOI:** 10.3390/diagnostics15141798

**Published:** 2025-07-16

**Authors:** Chang-Soo Yun, Chul-Ho Sohn, Jehyeong Yeon, Kun-Jin Chung, Byong-Ji Min, Chang-Ho Yun, Bong Soo Han

**Affiliations:** 1 Department of Radiation Convergence Engineering, College of Software and Digital Healthcare Convergence, Yonsei University, Wonju 26493, Republic of Korea; 2 Department of Radiology, Seoul National University College of Medicine, Seoul National University Hospital, Seoul 03080, Republic of Korea; 3 Department of Neurology, Seoul National University College of Medicine, Seoul National University Bundang Hospital, Seongnam 13620, Republic of Korea

**Keywords:** sleep, glymphatic system, diffusion tensor imaging analysis along the perivascular space index (DTI-ALPS) index

## Abstract

**Background/Objectives**: The glymphatic system plays a crucial role in clearing brain metabolic waste, and its dysfunction has been correlated to various neurological disorders. The Diffusion Tensor Imaging Analysis Along the Perivascular Space (DTI-ALPS) index has been proposed as a non-invasive marker of glymphatic function by measuring diffusivity along perivascular spaces; however, its sensitivity to sleep-related changes in glymphatic activity has not yet been validated. This study aimed to evaluate the feasibility of using the DTI-ALPS index as a quantitative marker of dynamic glymphatic activity during sleep. **Methods**: Diffusion tensor imaging (DTI) data were obtained from 12 healthy male participants (age = 24.44 ± 2.5 years; Pittsburgh Sleep Quality Index (PSQI) < 5), once while awake and 16 times during sleep, following 24 h sleep deprivation and administration of 10 mg zolpidem. Simultaneous MR-compatible electroencephalography was used to determine whether the subject was asleep or awake. DTI preprocessing included eddy current correction and tensor fitting. The DTI-ALPS index was calculated from nine regions of interest in projection and association areas aligned to standard space. The final analysis included nine participants (age = 24.56 ± 2.74 years; PSQI < 5) who maintained a continuous sleep state for 1 h without awakening. **Results**: Among nine ROI pairs, three showed significant increases in the DTI-ALPS index during sleep compared to wakefulness (Friedman test; *p* = 0.027, 0.029, 0.034). These ROIs showed changes at 14, 19, and 25 min after sleep induction, with FDR-corrected *p*-values of 0.024, 0.018, and 0.018, respectively. **Conclusions**: This study demonstrated a statistically significant increase in the DTI-ALPS index within 30 min after sleep induction through time-series DTI analysis during wakefulness and sleep, supporting its potential as a biomarker reflecting glymphatic activity.

## 1. Introduction

Sleep is an important physiological process that regulates the immune system, cognitive abilities, neurological homeostasis, and overall health [1,2,3]. Iliff et al. reported that cerebrospinal fluid (CSF) facilitates the removal of extracellular solutes, including neurotoxic proteins, from the mouse brain [4]. This mechanism, termed the glymphatic system, involves CSF flow parallel to blood flow within the perivascular space (PVS), driven by arterial wall pulsation [4,5]. The aquaporin-4 water channel, prominently polarized in the astrocyte endfeet, facilitates CSF entry into the brain parenchyma [4,5]. Aquaporin-4 also enables the exchange of interstitial fluid (ISF), promoting the clearance of extracellular solutes and neurotoxic proteins such as beta-amyloid [4]. Glymphatic activity increases during sleep and is accompanied by an expansion of the interstitial space [6]. This expansion facilitates the convective exchange of CSF and ISF, thereby enhancing the clearance of neurotoxic proteins compared to the awake state. Thus, evaluating glymphatic activity during sleep is important for diagnosing sleep-related diseases and neurodegenerative disorders [7,8,9]. Although animal studies have provided insights into the glymphatic system, assessing its function in the human brain remains challenging due to the limited availability of non-invasive evaluation methods. Glymphatic dysfunction is increasingly recognized as a contributing factor in the pathogenesis of various neurodegenerative diseases; however, the lack of clinically applicable, non-invasive imaging biomarkers continues to impede early diagnosis, therapeutic intervention, and long-term monitoring of treatment efficacy.

Recent advances in magnetic resonance imaging (MRI) techniques have enabled the non-invasive evaluation of the glymphatic system in the human brain [10,11,12]. Contrast-enhanced MRI with gadolinium-based contrast agents (GBCAs) has demonstrated that putative glymphatic activity increases during sleep relative to the awake state [13]. However, intrathecal administration of GBCAs has several limitations, including adverse effects of contrast agents, such as procedural discomfort associated with lumbar puncture and the need to deliver a high concentration of potentially toxic agents [12,14]. In addition, GBCA-based methods are limited in observing immediate dynamic glymphatic activity due to issues such as contrast agent recirculation, delayed signal onset, and physiological confounds related to systemic organ function [15]. For this reason, diffusion tensor imaging (DTI) has been widely used as a non-invasive method to assess glymphatic activity in the human brain [16,17,18,19]. Among the DTI-based approaches, Taoka et al. introduced the Diffusion Tensor Imaging Analysis Along the Perivascular Space (DTI-ALPS) index as a specific metric to quantify diffusivity along the PVS, serving as an indicator of glymphatic activity [19].

The medullary veins are primarily aligned in the left–right direction (e.g., the *x*-direction), perpendicular to the ventricular body in the brain [20]. PVS flows parallel to these medullary veins between subcortical fibers, draining the solutes and neurotoxic proteins into the brain parenchyma and facilitating the clearance of interstitial fluid from the brain [19,20,21]. The directional alignment of the medullary veins causes asymmetry in radial diffusion coefficients measured from nerve fibers that cross these veins perpendicularly. Specifically, projection and association fibers that intersect the veins at right angles exhibit different radial diffusion properties depending on their orientation relative to the medullary veins [19]. This asymmetry is quantified by the DTI-ALPS index and is attributed to the *x*-directional arrangement of the PVS. Thus, a low DTI-ALPS index suggests reduced water movement within the PVS between the medullary veins, indicating potential dysfunction of glymphatic activity [22,23]. As previous studies using the DTI-ALPS index have predominantly focused on comparisons between healthy controls and individuals with neurological disorders during the awake state, the relationship between the DTI-ALPS index and glymphatic activity remains insufficiently validated [19,23,24].

This study aimed to evaluate the feasibility of the DTI-ALPS index as a quantitative biomarker for assessing glymphatic activity during sleep. Regions of interest (ROIs) were optimized within the left superior corona radiata and superior longitudinal fasciculus based on diffusion coefficient analysis. Using these ROIs, temporal variations in the DTI-ALPS index were assessed during sleep states over a period of 1 h. In addition, the individual components of the DTI-ALPS index were analyzed to assess their contributions to glymphatic activity.

## 2. Materials and Methods

### 2.1. Study Design

This study received ethical approval from the Yonsei University MIRAE Campus Institutional Review Board (1041849-202305-BM-089-10). All participants were recruited according to exclusion criteria, which ruled out individuals with a history of neurological or psychiatric conditions, night shift work, or heavy smoking (more than five cigarettes per day) [18]. All initially recruited participants also had Pittsburgh Sleep Quality Index (PSQI) scores below five and a regular sleep pattern with sleep efficiency greater than 90% at the time of enrollment [18,25]. To minimize age-related variability and facilitate clearer observation of glymphatic activity, only healthy male participants in their 20s, an age group expected to exhibit optimal glymphatic function, were included in this study. A total of 20 participants were initially recruited. However, eight participants were excluded prior to DTI scans due to newly discovered claustrophobia or personal requests to terminate the examination. Thus, 12 participants who completed the imaging acquisition protocol were included in the study (mean age: 24.44 ± 2.5 years; gender: male; PSQI score < 5; average sleep efficiency: 92.04%). All participants provided written informed consent. Subsequently, each participant underwent 24 h of sleep deprivation, during which two researchers monitored their wakefulness every 30 min via phone calls and text messages to ensure compliance. EEG data were obtained to verify the sleep states of participants.

All experiments were scheduled to begin at 22:00 to minimize potential confounding factors that could affect physiological measurements. An EEG cap with 32 electrodes, a BrainAmp EEG amplifier, and Brain Vision EEG Recorder software (Version 1.23.0003; Brain Products GmbH, Munich, Germany) were used to verify sleep states between DTI scans. All participants consumed 10 mg of zolpidem 30 min before the first DTI scan. A 10 mg dose of zolpidem is a standard prescription for adults, and this short-acting hypnotic drug reaches its peak concentration in the bloodstream approximately 45 min after administration [26]. After the administration of zolpidem, participants underwent the first DTI scan during the awake state to establish a baseline for the DTI-ALPS index (22:21:57 ± 0:07:01). The interval between zolpidem intake and the awake DTI scan was 16:49 ± 06:21 min. After the awake DTI scan, participants were instructed to sleep in preparation for the subsequent DTI scans during the sleep state (22:31:38 ± 0:00:58). Susceptibility-weighted imaging (SWI) was acquired for 5 min to identify the location of the medullary veins, which is necessary for calculating the DTI-ALPS index. Subsequently, 17 additional DTI scans were obtained during the participants’ sleep states to evaluate glymphatic activity. Each DTI scan was conducted at 90 s intervals to allow EEG data acquisition without diffusion gradient artifacts. After the DTI scans during sleep, T1-weighted imaging was obtained.

**Figure 1:**
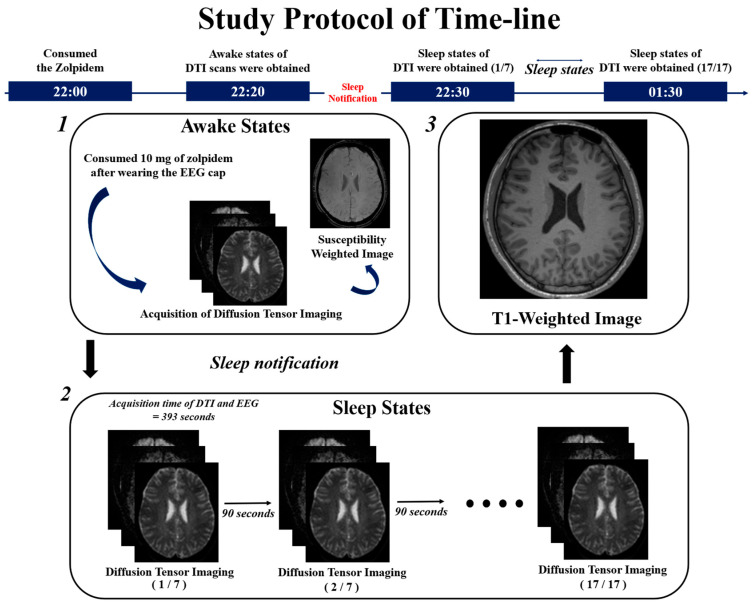
Experimental protocol timeline for assessing glymphatic activity during sleep states. (**1**) Initial acquisition of diffusion tensor imaging (DTI) is followed by susceptibility-weighted imaging in the awake state. (**2**) Time-series DTI scans are obtained at 90 s intervals to allow electroencephalogram acquisition without diffusion gradient artifacts. (**3**) A T1-weighted image is acquired to localize the anatomical structures of the brain in each participant.

The sleep states of all participants were assessed using EEG recordings acquired at 90 s intervals between DTI scans. Three independent sleep EEG experts, each with over 10 years of experience, evaluated the EEG data in a blinded manner to ensure unbiased judgment. To ensure the reliability of the analysis, only EEG data for which at least two out of three experts provided consistent evaluations of the participant’s sleep state were included, and datasets with complete disagreement among the experts were excluded. Finally, 9 participants (mean age = 24.56 ± 2.74 years, male; average sleep efficiency = 93.98%; PSQI score < 5) who maintained stable sleep and did not awaken for 1 h during the DTI scans were included in the analysis of the DTI-ALPS index. Figure 1 illustrates the experimental protocols employed in this study.

### 2.2. Image Acquisition Protocol

A 3T MRI scanner (SIEMENS MAGNETOM Skyra; Siemens Healthcare, Erlangen, Germany) with a 32-channel brain coil was used for all image acquisitions. First, all DTI scans were obtained using a multi-slice single-shot diffusion-weighted echo-planar imaging with two shells (*b* = 800, 2800 s/mm^2^), sampled in 30 gradient directions each; field of view = 224 × 224 mm^2^, matrix size = 112 × 112, TR = 3800 ms, TE = 105 ms, multiband factor = 2, slice thickness = 2 mm without slice gap, eight *b* = 0 s/mm^2^ images. Additionally, a DTI scan was performed with posterior-to-anterior phase encoding, and *b* = 0 s/mm^2^ images were acquired to calculate the susceptibility-induced off-resonance field. Second, SWI was obtained using the following parameters: a three-dimensional gradient-recalled echo sequence, TR = 28 ms, TE = 20 ms, flip angle = 15°, matrix size = 269 × 269, and slice thickness = 2 mm without slice gap. Third, a T1-weighted image was obtained using the following parameters with MPRAGE sequence: TR = 230 ms, TE = 2.32 ms, flip angle = 9°, matrix size = 256 × 256, slice thickness = 0.9 mm. All axial images were acquired parallel to the anterior commissure–posterior commissure (AC–PC) line, as defined in the midsagittal plane.

### 2.3. Electroencephalography Analysis

**Figure 2:**
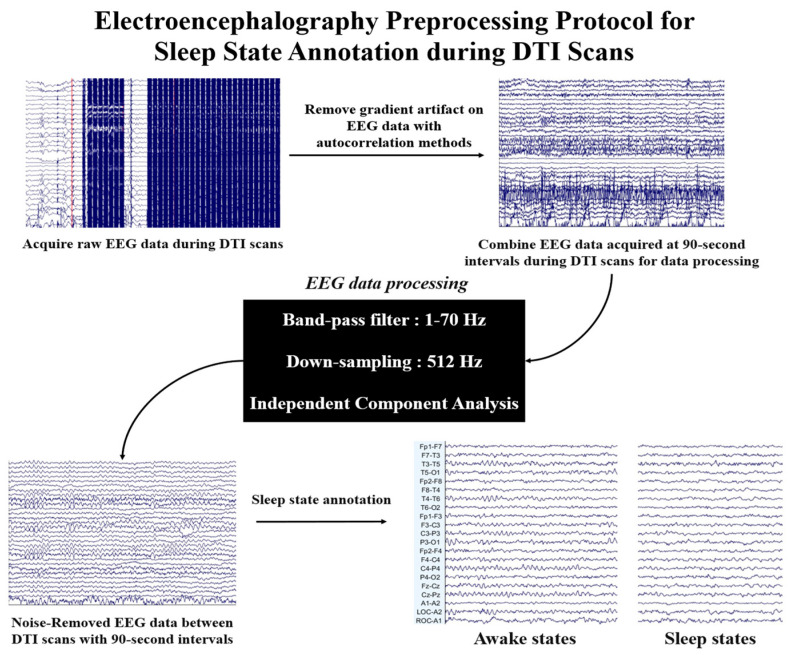
Electroencephalogram (EEG) data preprocessing protocol for identifying the sleep states of participants. Raw EEG data were acquired during diffusion tensor imaging (DTI) scans. Gradient artifacts in the EEG data were removed using autocorrelation methods. After artifact removal, EEG data obtained at 90 s intervals during DTI scans were combined for further processing. The preprocessing steps included applying a band-pass filter (1–70 Hz), down-sampling to 512 Hz, and performing independent component analysis.

EEG data were obtained at a high sampling rate of 5000 Hz, using a band-pass filter set between 0.1 to 250 Hz. During MRI scans, the impedance between electrodes was maintained below 5 kΩ to ensure participants’ safety [27]. To ensure precise identification of sleep states, autocorrelation analysis is applied to the EEG data to detect and exclude segments influenced by gradient artifacts [27]. This approach allows for the integration of artifact-free EEG data recorded during the 90 s intervals between DTI scans, thereby maintaining the reliability and accuracy of the analysis [28]. After collecting the 90 s data, all EEG data are further processed, including the application of a 1–70 Hz band-pass filter and down-sampling to 512 Hz. Cardiac pulse artifacts are removed using independent component analysis [29]. All EEG data processing is conducted using EEGLAB software (version 2020.0), a widely respected toolbox for EEG data analysis developed based on MATLAB (R2023a, Natick, MA, USA: The MathWorks Inc.) [30]. Following preprocessing of EEG data, sleep states are classified based on established neurophysiological criteria. Participants are assumed to be asleep during a scan if they are verified to be asleep immediately before and after the DTI scan. Sleep stages were classified according to the criteria established by the American Academy of Sleep Medicine (AASM), including N1, N2, N3, and Rapid Eye Movement (REM). Stage classification was performed by three independent EEG experts based on characteristic neurophysiological features such as sleep spindles, K-complexes, and delta wave activity [31]. In cases where precise classification into specific sleep stages was not feasible due to residual artifacts or insufficient characteristic features, the corresponding EEG segments were evaluated based on expert consensus and categorized as either wakefulness or sleep. EEG signals were scored in 90 s epochs as either 0 (awake) or 1 (sleep). This binary classification approach was applied to ensure the inclusion of analyzable data while maintaining the integrity of the staging process. Figure 2 illustrates the EEG data preprocessing protocol along with annotations of awake and sleep states, as evaluated by three EEG experts.

### 2.4. Diffusion Tensor Imaging Preprocessing

**Figure 3:**
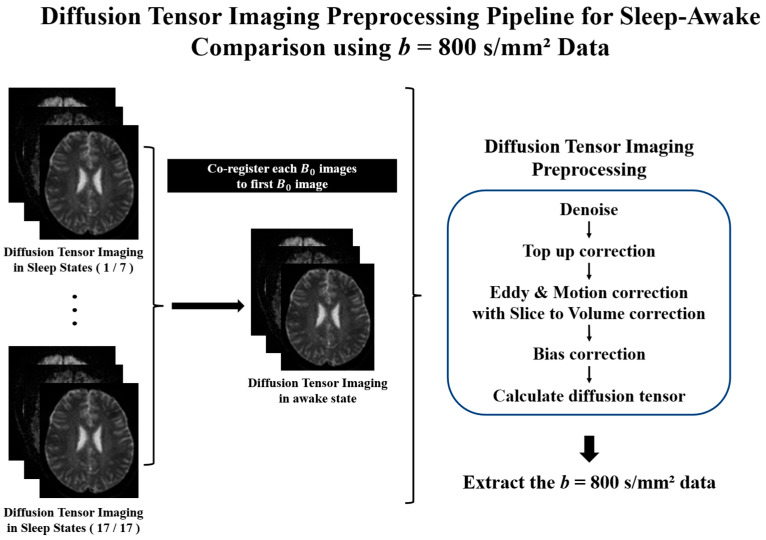
Preprocessing protocols for diffusion tensor imaging (DTI) to calculate the Diffusion Tensor Imaging Analysis Along the Perivascular Space (DTI-ALPS) index. All DTI scans are registered to the first image to minimize motion effects between scans. The registered DTI scans are performed to noise reduction, susceptibility distortion correction, eddy current correction, inter-volume motion correction, and bias field correction. The diffusion tensor is calculated using only the DTI volumes with a *b*-value of 800 s/mm^2^ following preprocessing.

All DTI data are preprocessed using the FSL software library (version 6.0.1) and MRtrix3 (version 3.0.3). To minimize motion effects between DTI scans, sleep DTI data are registered to the awake state using a rigid-body transformation. After preprocessing the DTI data, we perform noise removal, correction for Gibbs ringing artifacts, adjustment for susceptibility-induced distortions, correction for eddy current-induced distortions, and correction for motion between DTI volumes using slice-to-volume correction [32,33,34,35]. Following DTI preprocessing, we calculate the diffusion coefficients along with the *x*-, *y*-, and *z*-axes for each DTI dataset. Since previous studies have demonstrated that slow and fast apparent diffusion coefficients derived from low *b*-values provide a more accurate reflection of glymphatic system functionality, we focused our analysis on these parameters [16]. Finally, this study uses only *b*-values of 800 s/mm^2^ to calculate the DTI-ALPS index. Figure 3 illustrates the preprocessing protocols used to calculate the DTI-ALPS index in this study.

### 2.5. The DTI-ALPS Index with Perivascular Space

**Figure 4:**
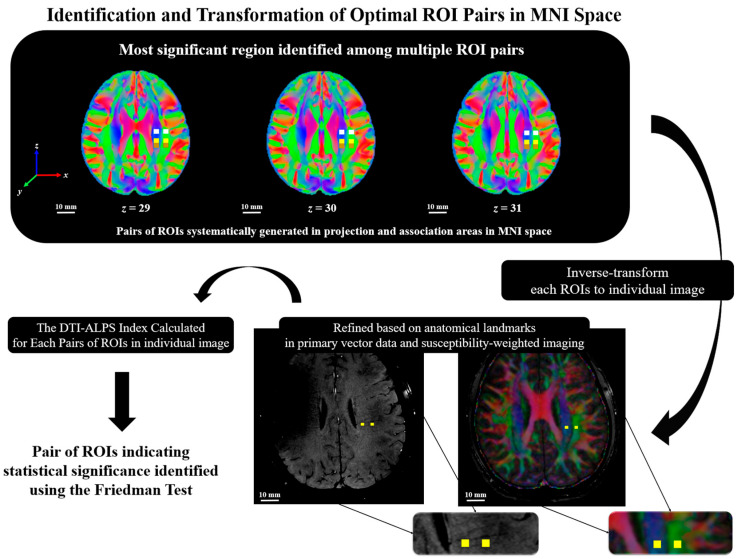
The complete analysis pipeline, including ROI placement and refinement within the MNI space, the application of statistical methods, and the identification of the optimal ROI pair for further analysis. Initially, multiple ROI pairs are systematically defined within the projection and association fiber regions in the MNI space, specifically on axial slices z = 29, 30, and 31. Each ROI measures 3 mm × 3 mm (corresponding to 3 × 3 voxels at 1 mm isotropic resolution), centered at predefined coordinates aligned with major fiber directions. These ROIs are subsequently transformed into each subject’s native diffusion space using inverse warping. In the native space, anatomical refinement is performed based on individual primary diffusion vector maps and susceptibility-weighted images to ensure accurate placement along perivascular structures. Following this refinement, the Diffusion Tensor Imaging Analysis Along the Perivascular Space (DTI-ALPS) index is computed for each ROI pair and subject. Finally, a Friedman test is applied across time points to identify ROI pairs that exhibit statistically significant temporal changes in the DTI-ALPS index, reflecting potential regional glymphatic activity. The white, yellow, and brown box pairs indicate the ROIs located at *y* = −17, −27, and −29, respectively.

To ensure consistent and reproducible analysis in calculating the DTI-ALPS index, all ROIs are defined using the Montreal Neurological Institute (MNI) template. Specifically, each ROI is positioned as a 3 mm × 3 mm rectangular area within the left hemisphere of the MNI space, utilizing the primary eigenvector (V1) map with 1 mm isotropic spatial resolution. Each pair of ROIs is strategically placed in the projection and association fiber regions, aligned parallel to the subcortical fiber tracts as described by Taoka et al. [19]. In this study, nine pairs of ROIs are systematically placed across three axial slices located at *z* = 29, 30, and 31, respectively, with each slice containing three ROI pairs, as illustrated in Figure 4. The three ROI pairs in each axial slice are positioned with centers at (*x*, *y*) = (−26, −17) and (−37, −17), (−26, −27) and (−37, −27), and (−26, −29) and (−37, −29) in the projection and association areas, respectively, from front to back.

The resulting nine ROI pairs delineated in the MNI space are transformed into each subject’s native diffusion space using inverse transformations derived from the registration process. To improve anatomical precision, each ROI is redefined as a 2 mm × 2 mm square within the original ROI area. ROIs are visually inspected for proper alignment with the white matter tracts and medullary veins. Those with clear misalignment are excluded. ROIs with minor misalignment are adjusted within a ±1 mm range to optimize overlap with the target tract and ensure intersection with medullary veins. The DTI-ALPS index is calculated using the defined ROIs in individual space, according to Equation (1) [19]:

(1) $$DTI-ALPS\;index=\frac{mean({Dxx}_{proj},\;{Dxx}_{asso})}{mean({Dyy}_{proj},\;{Dzz}_{asso})}$$

${Dxx}_{proj}$ and ${Dxx}_{asso}$ indicate diffusivity along the *x*-axis in the projection and association areas, respectively. ${Dyy}_{proj}$ and ${Dzz}_{asso}$ indicate diffusivity along the *y*- and *z*-axis (perpendicular directions) in the projection and association areas, respectively. Multiple ROI pairs are evaluated across the MNI space to identify regions suitable for the DTI-ALPS index calculation. ROI pairs that exhibit statistically significant differences between the awake and sleep states and show increases in the index primarily due to enhanced diffusivity along the *x*-axis, which reflects water movement through the PVS, are selected for further analysis.

### 2.6. Statistical Analysis

Figure 4 illustrates the overall analysis pipeline, including ROI selection and refinement in the MNI space, the statistical procedures used to identify significant temporal changes, and the final determination of the optimal ROI pair for subsequent analysis. All statistical analyses are performed using MATLAB (R2023a, The MathWorks Inc., Natick, MA, USA). To assess differences in the DTI-ALPS index across repeated DTI scans, the Friedman test, which is a non-parametric test suitable for repeated measures, is applied. The Friedman test, a suitable non-parametric method for one-way repeated measures, is chosen due to the small sample size and non-normal distribution of the data, which violates the assumptions of parametric tests. To control Type I errors associated with multiple pairwise comparisons, the significance threshold is set at *p* < 0.05 with false discovery rate (FDR) correction.

## 3. Results

**Figure 5:**
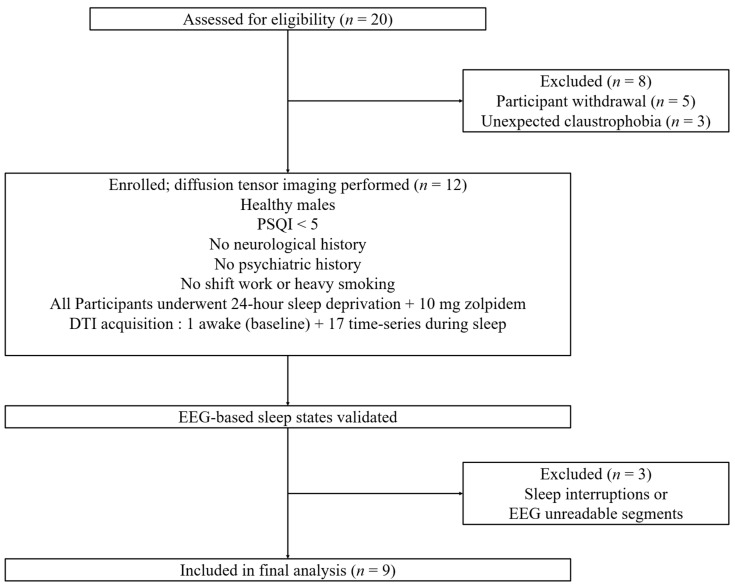
Flowchart of participant selection, data acquisition, and exclusion steps. A total of 20 participants were screened; 12 completed diffusion tensor imaging (DTI) following sleep deprivation and zolpidem administration. After EEG-based sleep state validation and exclusion of data with motion artifacts, 9 were included in the final analysis.

This study assessed glymphatic activity using the DTI-ALPS index, beginning with measurements taken during the awake state and followed by continuous data acquisition over an approximately one-hour sleep period. DTI data was initially obtained from twelve participants, but datasets exhibiting excessive motion artifacts during scanning—which rendered reliable analysis infeasible—were excluded from further analysis. As a result, temporal changes in the DTI-ALPS index were examined in nine participants. To further characterize the sleep state during DTI data acquisition, sleep annotations were generated based on EEG recordings. The overall process of participant recruitment, inclusion, MRI acquisition, EEG-based sleep state validation, and exclusion is summarized in Figure 5. Among the 20 individuals initially assessed for eligibility, 12 participants who met the inclusion criteria underwent DTI scanning following 24 h of sleep deprivation and administration of 10 mg zolpidem. Subsequent EEG-based validation identified three datasets as unsuitable for final analysis due to sleep interruptions or unreadable EEG segments. Accordingly, the final analysis was conducted on data from nine participants who maintained stable sleep throughout the scanning period.

Table 1 summarizes the sleep states of the twelve participants during the approximately two-hour DTI acquisition. Sleep states were annotated based on EEG recordings at each time point during the scanning session to track transitions between the awake state and sleep stages. To assess inter-rater reliability, Fleiss’ Kappa was calculated across all annotations, yielding a value of κ = 0.812, which indicates a high level of agreement among raters. These annotations provided a temporal reference for identifying periods of stable sleep state. However, due to residual gradient and cardiac-related artifacts inherent to simultaneous EEG and MRI acquisition, it was not possible to reliably distinguish detailed sleep stages such as N1, N2, N3, and REM. As a result, the analysis was limited to binary classification between wakefulness and sleep states.

**Figure 6:**
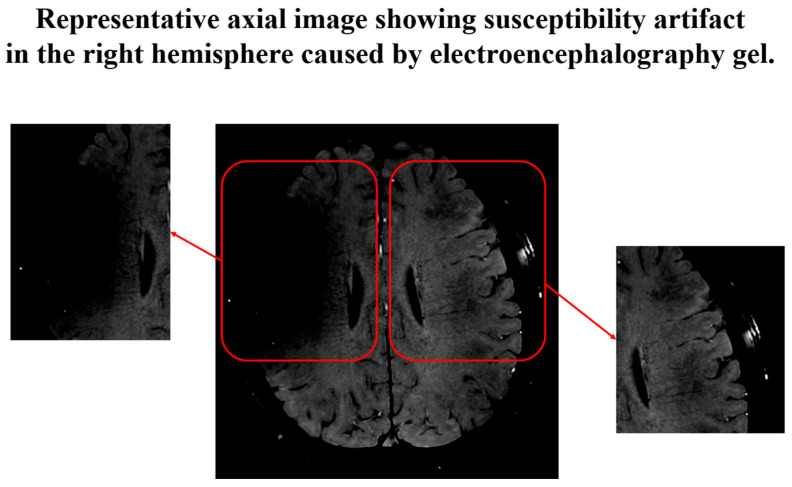
Susceptibility artifact caused by electroencephalography (EEG) gel application, as observed in a representative axial slice. The red boxes highlight bilateral regions of interest (ROIs), with the right hemisphere exhibiting signal dropout and geometric distortion due to susceptibility effects. These artifacts can compromise diffusion metric reliability, supporting the exclusion of the right hemisphere in subsequent analyses.

In addition, to eliminate the influence of artifacts caused by EEG gel, the right hemisphere, where susceptibility artifacts observed in SWI impeded clear visualization of the deep medullary veins, was excluded from the DTI ALPS index analysis. A representative image was included in Figure 6 to illustrate this issue. Based on these observations, the analysis was limited to the left hemisphere, where image quality remained stable and reliable across all participants.

**Figure 7:**
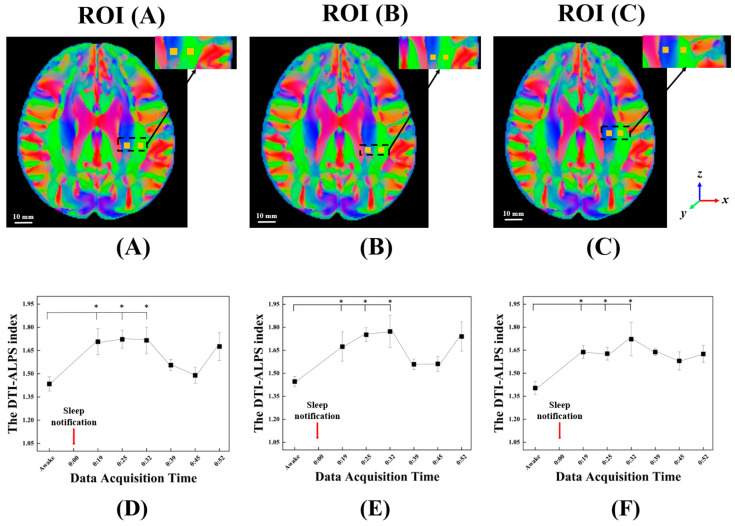
ROI pairs used for the Diffusion Tensor Imaging Analysis Along the Perivascular Space (DTI-ALPS) index. The DTI-ALPS index analyses and their temporal trajectories during sleep. The upper panels (**A**–**C**) depict the locations of the three representative ROI pairs overlaid on the color-coded standard template. The lower panels (**D**–**F**) show the corresponding temporal changes in the DTI-ALPS index for each ROI pair. The red vertical line indicates the onset of sleep notification, which occurred approximately 9 min after the awake scan. Asterisks (*) denote timepoints at which the DTI-ALPS index significantly increased compared to the awake state, based on FDR-adjusted post hoc comparisons (adjusted *p* = 0.024, 0.018, and 0.018). (**A**) ROI pair (yellow boxes): projection (−26, −27, 29); association (−37, −27, 29). This ROI showed the most prominent increase, with significant elevations in both *x*-directional diffusivity and the DTI-ALPS index (*p* = 0.027), as shown in (**D**). (**B**) ROI pair (yellow boxes): projection (−26, −29, 29); association (−37, −29, 29). This pair demonstrated a statistically significant increase in the DTI-ALPS index (*p* = 0.029) (**E**), although changes in *x*-directional diffusivity were less pronounced. (**C**) ROI pair (yellow boxes): projection (−26, −17, 29); association (−37, −17, 29). This region also exhibited a significant increase in the DTI-ALPS index (*p* = 0.034) (**F**).

Among the nine initially defined ROI pairs in left hemisphere, four were excluded due to misalignment with white matter tracts or medullary veins. An additional two pairs were excluded because they did not exhibit statistically significant changes in the DTI-ALPS index during sleep. Consequently, the remaining three ROI pairs, which demonstrated significant changes between the awake and sleep states, were further analyzed using the Friedman test, as illustrated in Figure 7.

ROI (A), with projection coordinates at (−26, −27, 29) and association coordinates at (−37, −27, 29), exhibited the most robust response, showing significant increases in both *x*-directional diffusivity and the DTI-ALPS index (*p* = 0.027; Figure 7D). ROI (B), located at (−26, −29, 29) for the projection and (−37, −29, 29) for the association area, also showed a significant increase in the DTI-ALPS index (*p* = 0.029; Figure 7E). ROI (C), with projection and association coordinates at (−26, −17, 29) and (−37, −17, 29), respectively, demonstrated a significant increase in the DTI-ALPS index as well (*p* = 0.034; Figure 7F).

The relative increase in the DTI-ALPS index was calculated by comparing the peak time point during sleep with the baseline measured in the awake state for each ROI pair. ROI (A) increased from 1.43 to a peak value of 1.71, representing a 20.80% rise, with a significant increase observed between 14 and 25 min after sleep notification. ROI (B) showed a comparable increase from 1.42 to 1.71 (20.79%), reaching its peak at 25 min, with significant changes observed during the same interval. ROI (C) increased from 1.39 to 1.70, corresponding to a 22.60% rise, with a clear upward trend beginning at 10 min and peaking at 25 min.

To further identify the specific time points contributing to the observed group differences, post hoc pairwise comparisons were performed for ROI (A). Following FDR correction, all three time points remained statistically significant, with adjusted *p*-values of 0.024, 0.018, and 0.018 at approximately 14, 19, and 25 min after sleep notification, regardless of ROI. The increases in the DTI-ALPS index at these points corresponded to approximately a 20% elevation relative to the awake state. In addition to statistical significance, effect sizes (Wilcoxon’s r) were calculated for each comparison to quantify the magnitude of change. Large effect sizes were observed at these early time points, further supporting the physiological relevance of DTI-ALPS index elevations during sleep onset. The results, adjusted for multiple comparisons using the FDR method, including effect sizes, are summarized in Table 2.

To identify the factors contributing to changes in the DTI-ALPS index, its numerator and denominator components were analyzed, as shown in Table 3. The *x*-directional diffusivity, $mean({Dyy}_{proj},\;{Dzz}_{asso})$ increased by 10.57% to 25.46 min after sleep notification.

In contrast, the perpendicular diffusivity components (e.g., $mean({Dyy}_{proj},\;{Dzz}_{asso})$) showed a maximum decrease of 14.32% at 19.46 min. The DTI-ALPS index exhibited its greatest increase at 19.46 min, rising by 20.81% relative to the awake state. Table 3 summarizes the rate of change in both the numerator and denominator of the DTI-ALPS index.

## 4. Discussion

This study investigated whether glymphatic activity could be evaluated by analyzing temporal changes in the DTI-ALPS index during the transition from wakefulness to sleep in healthy participants who were administered the hypnotic agent zolpidem.

Our results showed that in three ROIs—(A), (B), and (C)—the DTI-ALPS indices exhibited statistically significant increases at approximately 14 min, 19 min, and 25 min after following sleep notification, compared to the awake state. As illustrated in Figure 7, all three ROIs exhibited a decrease in the DTI-ALPS index to 31 min and then demonstrated a rebound at 43 min. Although the reason for the slight decrease in the DTI-ALPS index after 31 min remains unclear, it is presumed that transitional changes in sleep state may have contributed to this pattern. ROI (A) and ROI (B) exhibited nearly identical patterns of change, likely attributable to their anatomical adjacency along the *y*-axis. The temporal pattern observed in ROI (C) was largely consistent with that of ROI (A) and ROI (B), albeit with some differences. Accordingly, the subsequent discussion will focus on the results observed in ROI (A).

In ROIs within projection fibers oriented along the z-axis and association fibers oriented along the *y*-axis, radial diffusivity is influenced by multiple brain tissue components, including neural fiber structures, medullary vessels, and PVS [19,20,21]. The DTI-ALPS index reflects the anisotropy between ${Dxx}_{proj}$ and ${Dyy}_{proj}$ in the projection area, and ${Dxx}_{asso}$ and ${Dzz}_{asso}$ in the association area. This anisotropy is primarily attributed to the orientation of deep medullary veins and their accompanying PVS, which predominantly align along the x-axis [21]. With increased glymphatic activity during sleep, CSF flow is expected to increase primarily in the *x*-direction, leading to an anticipated rise in the DTI-ALPS index.

As shown in Table 3, the average *x*-directional diffusivity, $mean({Dxx}_{proj},\;{Dxx}_{asso})$, increases by between 3.51% and 10.57% across all time points, with a maximum at 25 min. Conversely, the perpendicular diffusivity, $mean({Dyy}_{proj},\;{Dzz}_{asso})$ demonstrated decreases by between 14.32% and 3.35%, except for a 1.74% increase observed at 37 min. Since the rate of change in the DTI-ALPS index can be approximated as the rate of change in the $mean({Dxx}_{proj},\;{Dxx}_{asso})$ minus that in the $mean({Dyy}_{proj},\;{Dzz}_{asso})$, the observed percent changes at 25 min—20.23% for the DTI-ALPS index, 10.57% for $mean({Dxx}_{proj},\;{Dxx}_{asso})$, and −8.04% for the $mean({Dyy}_{proj},\;{Dzz}_{asso})$—suggest that their contributions to the DTI-ALPS index are comparable. In particular, at 14 min and 25 min, the $mean({Dyy}_{proj},\;{Dzz}_{asso})$ contributes more significantly to the DTI-ALPS index than the $mean({Dxx}_{proj},\;{Dxx}_{asso})$.

These findings challenge the prevailing assumption that increased *x*-directional diffusivity during sleep, attributed to enhanced glymphatic activity, is the primary contributor to the elevated DTI-ALPS index. Although the observed reduction in perpendicular diffusivity during sleep may result from sleep-related physiological changes, it has been presumed to be independent of glymphatic activity. Consequently, this raises concerns about the validity of the DTI-ALPS index as a specific marker of glymphatic function. This prompts an important question: What, then, is the underlying cause of the reduction in vertical diffusivity during sleep? When glymphatic activity increases during sleep, the following physiological changes are anticipated: (i) increased CSF influx into the brain parenchyma, accompanied by expansion of the extracellular space [6]; (ii) potential changes in medullary vein diameter and PVS width [36]; and (iii) enhanced glymphatic flow within the PVS, facilitating the clearance of ISF containing metabolic waste.

The increase of CSF influx into the brain parenchyma, accompanied by expansion of the extracellular space, is likely to exert minimal influence on the anisotropy between radial diffusivities, as the expansion of extracellular space is isotropic in nature [37]. Additionally, in rodents, sleep has been reported to be associated with a decrease in PVS width and an increase in arteriole diameter [36]. Although such changes were observed in the arteriole and have not yet been demonstrated in humans, the changes in medullary vein diameter and PVS width may also occur. However, their magnitudes are expected to be minimal, and the net change in perpendicular diffusivity is likely to be negligible [36,37,38]. Thus, the only remaining factor likely to influence the DTI-ALPS index appears to reflect glymphatic activity. During wakefulness, CSF flow is relatively suppressed [39], and the perivenous space may present a more complex and disorganized structure. This complexity likely causes glymphatic flow to include both axial (along the vessel) and non-axial (including perpendicular) diffusivity components [40,41,42]. In contrast, during sleep, glymphatic activity increases, and the PVS structure may be reorganized to support a more streamlined, unidirectional flow aligned with the medullary vein axis [41]. This reorientation may reduce the perpendicular component of CSF movement within the PVS, thereby decreasing the measured perpendicular diffusivity, despite the overall increase in flow. These results suggest that the reduction in perpendicular diffusivity during sleep reflects not a decrease in pure diffusion capacity, but rather a reorganization of glymphatic activity toward more efficient axial clearance along perivenous pathways. Consequently, the observed decrease in perpendicular diffusivity may also be attributed to enhanced glymphatic activity. Taken together, since both the increase in *x*-directional diffusivity and the decrease in perpendicular diffusivity are likely consequences of increased glymphatic activity, the DTI-ALPS index appears to be a suitable indicator for evaluating glymphatic activity.

Despite the promising results, several limitations should be acknowledged. First, the inclusion of only healthy young male participants limits the generalizability of the present findings. Glymphatic activity is influenced by age, sex, and overall health status; therefore, future studies should aim to recruit larger and more demographically diverse cohorts, including females, older adults, and individuals with neurodegenerative diseases, to establish the reliability and clinical relevance of the DTI-ALPS index across broader populations. Second, in a pilot study conducted by our research team without the use of sleep aids, none of the participants were able to maintain a stable sleep state for one hour. For this reason, zolpidem was administered in the present study to ensure stable sleep throughout the imaging acquisition. While this approach enables successful data collection, pharmacologically induced sleep may not fully replicate the physiological characteristics of natural sleep, particularly in terms of cerebrovascular dynamics [43,44,45]. Recent evidence from a human study by Hauglund et al. (2025) demonstrated that zolpidem suppresses cerebrovascular pulsatility, which is a primary driver of glymphatic clearance during sleep [46]. This suggests that zolpidem may impair glymphatic function by dampening the vascular mechanisms that facilitate interstitial fluid movement. Although zolpidem has been reported to preserve certain physiological characteristics of non-REM sleep, such as slow-wave activity, these do not fully capture its effects on cerebral hemodynamics. Therefore, the use of zolpidem represents a potential limitation of our study. Future investigations comparing zolpidem-induced and natural sleep conditions are essential to determine the physiological equivalence of glymphatic activity across sleep induction methods. Third, technical challenges related to simultaneous EEG and diffusion MRI acquisition affected data quality and interpretability. Magnetic susceptibility artifacts resulting from EEG gel application caused signal dropouts in the right hemisphere, particularly near the deep medullary veins. As a result, ROIs in the right hemisphere were excluded from the analysis, thereby limiting the spatial coverage of the DTI-ALPS index measurements to the left hemisphere. This hemispheric restriction should be considered when interpreting the results, as it may limit the generalizability of findings to bilateral brain function. To overcome these limitations, future studies should consider employing EEG systems that do not require conductive gel, such as gel-free or dry electrode systems, which can significantly reduce magnetic susceptibility artifacts in MR environments [47]. Additionally, using MR-compatible EEG caps with optimized lead placement or shielding may further minimize interference and improve EEG signal quality during simultaneous EEG-MRI acquisition. In addition, although it was possible to distinguish between wakefulness and sleep, the presence of large residual noise in the EEG signals—even after the removal of pulse artifacts—prevented the reliable identification of specific sleep stages such as N1, N2, and N3. This limitation hindered sleep stage-specific analysis [48,49]. To overcome this, it is necessary to develop and apply a more sophisticated cardiac pulse artifact removal algorithm. Consequently, because sleep staging could not be performed in real time during MRI acquisition, data analysis relied on elapsed time from sleep notification rather than on physiologically confirmed sleep onset. This prevented alignment of the imaging data with specific sleep durations or stages, thereby limiting the precision of temporal interpretations. The scanning procedure itself, which required extended immobility following sleep deprivation and administration of a sedative, may also raise concerns regarding participant burden. Although no adverse events occurred in this study, future research should evaluate the tolerability and feasibility of the protocol in older adults and clinical populations. Lastly, because this study employed a single-session design, it was not possible to evaluate the diurnal variation of the DTI-ALPS index or assess the cumulative effects of chronic sleep disruption or improvement. Future longitudinal studies involving repeated measurements across different times of day and over extended periods are needed to determine the temporal stability and physiological sensitivity of the DTI-ALPS index to both acute and long-term changes in sleep physiology.

This study aimed to identify the specific time point during sleep when a significant increase in the DTI-ALPS index occurs and to evaluate its validity as a non-invasive marker of glymphatic activity. The results support its potential as a surrogate indicator; however, further research is needed to optimize measurement conditions and develop protocols that eliminate the need for pharmacological sleep induction. Expanding the study population and incorporating sleep stage–synchronized analysis will be essential for understanding glymphatic dynamics more comprehensively. Clinically, the DTI-ALPS index offers a safe, contrast-free tool suitable for repeated use, making it valuable for early detection of glymphatic dysfunction, monitoring therapeutic effects, and tracking sleep-related physiological changes in both research and clinical settings.

## 5. Conclusions

In this study, we investigated the temporal dynamics of the DTI-ALPS index in healthy male participants while they were in a sleep state. Our findings revealed significant increases in the DTI-ALPS index at 14, 19, and 25 min after sleep induction. These results were derived from one of three ROI pairs that showed statistically significant differences between sleep and wakefulness based on the Friedman test (*p* = 0.027, 0.029, and 0.034). For the ROI with the strongest effect (*p* = 0.027), FDR-corrected *p*-values at 14, 19, and 25 min were 0.024, 0.018, and 0.018, respectively. While the mean diffusivity in the *x*-direction $mean({Dxx}_{proj},\;{Dxx}_{asso})$—a parameter typically associated with increased glymphatic activity—showed an upward trend, these changes were not statistically significant. Unexpectedly, we observed a decrease in the mean diffusivity perpendicular to the medullary veins (y-direction in projection areas and z-direction in association areas). This reduction in perpendicular diffusivity contributed comparably or even more to the observed increase in the DTI-ALPS index compared to the *x*-directional diffusivity. Although the underlying mechanism for the decrease in perpendicular diffusivity during sleep remains unclear, the decrease was assumed to result from the reorganization of internal PVS structures to facilitate CSF flow, which accompanies increased glymphatic activity.

In conclusion, the DTI-ALPS index may be a well-defined measure to quantify glymphatic activity. Thus, future research should aim to elucidate the hydrodynamic properties of glymphatic activity within the PVS to enhance our understanding of its role in brain clearance mechanisms during sleep.

## Figures and Tables

**Table 1:** Sleep state annotations for each participant across 17 sequential DTI acquisition time points during the approximately two-hour MRI scanning session. Sleep states were categorized as awake (0), sleep state (1), or unusable (N) based on EEG recordings. Among the 12 participants, 9 subjects (Subjects 1–9) who maintained predominantly stable awake or sleep states with minimal unreadable segments were included in the final Diffusion Tensor Imaging Analysis Along the Perivascular Space (DTI-ALPS) index. For these 9 subjects, a total of 7 DTI time points were used, as indicated by the red box. Participants with frequent unreadable segments (‘N’) or unstable sleep patterns were excluded from subsequent analysis.

Sleep State Annotations for Each Participant Across 17 DTI Acquisition Time Points																		
		1	2	3	4	5	6	7	8	9	10	11	12	13	14	15	16	17
	
Subject 1		0	1	1	1	1	1	1	N	N	1	1	1	1	1	1	1	N
Subject 2		0	1	1	1	1	1	1	1	1	1	N	N	N	N	N	N	N
Subject 3		0	1	1	1	1	1	1	1	N	N	N	N	N	1	1	1	N
Subject 4		0	1	1	1	1	1	1	1	1	1	1	1	1	1	1	1	1
Subject 5		0	1	1	1	1	1	1	1	1	1	1	1	1	1	1	1	1
Subject 6		0	1	1	1	1	1	1	1	1	N	0	N	0	0	N	0	1
Subject 7		0	1	1	1	1	1	1	1	1	1	1	1	1	1	1	1	1
Subject 8		0	1	1	1	1	1	1	1	1	1	N	1	1	1	1	1	1
Subject 9		0	1	1	1	1	1	1	1	N	N	N	N	N	N	1	1	1
Subject 10		0	0	0	0	N	0	N	N	0	0	0	0	0	N	1	1	1
Subject 11		0	N	N	1	1	N	1	1	1	1	N	1	1	N	N	N	N
Subject 12		0	N	1	1	1	0	N	N	N	0	N	N	1	1	1	1	N

**Table 2:** Temporal changes in the Diffusion Tensor Imaging Analysis Along the Perivascular Space (DTI-ALPS) index (also shown in Figure 7A) during the first hour following sleep notification. The Friedman test revealed a significant main effect of time (*p* = 0.027), and post hoc pairwise comparisons corrected using the false discovery rate identified significant increases at approximately 14, 19, and 25 min relative to the awake state (adjusted *p* = 0.024, 0.018, and 0.018, respectively), corresponding to 19–20% increases from baseline. Asterisks in the figure indicate statistical significance, where * denotes uncorrected *p* < 0.05 and ** denotes FDR-corrected *p* < 0.05. In addition, effect sizes (Wilcoxon’s r) are provided for each comparison, with large effects observed at these timepoints, further supporting the physiological relevance of early DTI-ALPS index elevation during sleep.

Time Since Sleep Notification (Min)	Average of the DTI-ALPS Index (Standard Error)	Increase Ratio	*p*-Value	Adj. *p*-Value	Effect Size (r)
Awake	1.43 ± 0.05	-	-	-	-
14.46	1.71 ± 0.08	19.53%	0.012 *	0.024 **	0.640
19.46	1.72 ± 0.06	20.80%	0.003 *	0.018 **	0.887
25.46	1.72 ± 0.09	20.23%	0.006 *	0.018 **	0.587
31.45	1.56 ± 0.04	9.60%	0.156	0.187	0.534
37.45	1.49 ± 0.05	4.57%	0.445	0.445	0.131
43.45	1.68 ± 0.09	17.52%	0.064	0.096	0.755

**Table 3:** Percent changes in *x*-directional diffusivity $mean({Dxx}_{proj},\;{Dxx}_{asso})$, perpendicular diffusivity $mean({Dyy}_{proj},\;{Dzz}_{asso})$, and the Diffusion Tensor Imaging Analysis Along the Perivascular Space (DTI-ALPS) index. The DTI-ALPS index is relative to the awake state at each time point following sleep notification. The *x*-directional diffusivity represents the average value across the projection and association areas, while the perpendicular diffusivity reflects the average of diffusivity in the projection and association areas.

	Time Points (min)	Awake	14.46	19.46	25.46	31.45	37.45	43.45
Percent Change in								
$mean({Dxx}_{proj},\;{Dxx}_{asso})$		0	6.33%	3.51%	10.57%	5.94%	6.39%	4.51%
$mean({Dyy}_{proj},\;{Dzz}_{asso})$		0	−11.05%	−14.32%	−8.04%	−3.35%	1.74%	−9.53%
The DTI-ALPS index		0	19.53%	20.81%	20.23%	9.61%	4.57%	15.52%

## Data Availability

The data presented in this study are available on request from the corresponding author. The data are not publicly available due to their use in ongoing research projects.
